# Evaluating transitional care practices for adolescents with chronic conditions in two Dutch university hospitals: A mixed-methods study

**DOI:** 10.1016/j.hctj.2026.100149

**Published:** 2026-07-24

**Authors:** Jobert Sturm, Myrthe Braunstahl, Heidi van Heijningen-Tousain, Marieke van Summeren, Jane Sattoe, Johanna C. Escher, AnneLoes van Staa

**Affiliations:** a Department of Paediatric Gastroenterology, Erasmus University Medical Center - Sophia Children’s Hospital, P.O. Box 2060, Rotterdam 3000 CB, the Netherlands; b Research Centre Innovations in Care, Rotterdam University of Applied Sciences, P.O. Box 25035, Rotterdam 3001 HA, the Netherlands; c Department of General Paediatrics, University Medical Center Utrecht – Wilhelmina Children’s Hospital, P.O. Box 85090, Utrecht 3508 AB, the Netherlands

**Keywords:** Transitional care, Healthcare transition, Adolescents and young adults, Paediatric to adult care, Implementation, Mixed-methods, On Your Own Feet

## Abstract

**Background:**

The transition from paediatric to adult healthcare represents a critical period for young people with chronic conditions. Although structured transitional care is recommended both nationally and internationally, its implementation remains inconsistent across healthcare settings. This study aimed to examine the implementation of transition interventions in two Dutch university hospitals and to identify determinants shaping their implementation in routine practice.

**Methods:**

This convergent mixed-methods study evaluated 50 medical specialties across paediatric and/or adult services. Ninety healthcare professionals participated. Data were collected through semi-structured interviews and a structured verbal questionnaire assessing the presence of key transition interventions across preparation, transfer, and post-transfer integration phases. Interview data were analysed thematically, questionnaire data descriptively, and integrated findings were used to assess adherence to principles of transitional care.

**Results:**

Implementation varied across specialties and phases of care. A transition coordinator was reported in 31% of medical specialties. Individual transition plans were reported in 31% of paediatric services and 18% of adult services. A warm handover was reported in 53% of specialties. Key facilitators included defined roles, cross-boundary collaboration, and organisational support. Barriers comprised limited resources, insufficient adult-care involvement, and a lack of routinised processes. Principle-based scoring indicated mid-range implementation across specialties in both hospitals (range 8–27 on an 8–32 scale; median 14.5, IQR 12–20).

**Conclusions:**

Transitional care was implemented inconsistently across medical specialties in both hospitals, with the largest gaps occurring after transfer to adult care. Stronger organisational support, explicit coordination, routinised paediatric–adult care collaboration, and shared accountability may improve consistency and developmental appropriateness.

## 1 Introduction

The transition from paediatric to adult healthcare is a critical developmental milestone for adolescents and young adults (AYAs) with chronic conditions.^1^, ^2^ Early definitions of transition emphasized a planned transfer from child-centred to adult-oriented services.^3^ Over time, transition has increasingly been framed as a broader developmental process in which AYAs gradually develop autonomy and self-management skills while navigating psychosocial and social challenges.^4^, ^5^, ^6^ Although effective transitional care can prevent loss to follow-up and adverse health outcomes, its implementation and sustainability in routine practice remain challenging.^7^, ^8^, ^9^, ^10^ Continuity of care can be difficult as paediatric and adult services often differ in routines, organisation, and expectations about patient autonomy. In addition, financing structures—and the lack of reimbursement for joint transition activities—may further limit paediatric–adult collaboration and impede sustained follow-up.^11^, ^12^, ^13^

International guidance increasingly stresses the need for structured, individualised, and developmentally appropriate transitional care. Core elements include early and gradual preparation, clear coordination between paediatric and adult services, effective transfer communication, and continued support after transfer to adult care.^12^, ^14^, ^15^ The English National Institute for Health and Care Excellence (NICE) guideline complements these recommendations by offering practical, service-level advice on implementation and by delineating roles and responsibilities across the transition pathway.^16^

To support implementation, several practice frameworks translate these principles into concrete elements of routine care. For example, the Got Transition programme offers structured tools encompassing transition policies, readiness assessment, individual transition planning, transfer communication, and confirmation of transfer completion.^17^, ^18^ In the United States, paediatric and adult professional societies, through consensus-based guidance, frame transitional care as a set of guiding principles for routine clinical practice, with explicit emphasis on shared accountability between paediatric and adult services.^19^ In practice, these frameworks are often adapted through locally developed improvement projects to align with specific organisational and clinical contexts.^20^

Despite this consensus and the availability of tools, empirical studies and systematic reviews demonstrate substantial variation in the delivery of transitional care across conditions and settings. Implementation efforts often focus on preparation within paediatric services, whereas cross-boundary coordination and post-transfer support in adult care are less consistently organised.^8^, ^21^, ^22^ Evaluation is also inconsistent, with limited routine use of validated patient-reported outcome measures (PROMs) and variable assessment of transition experiences.^21^, ^23^ Moreover, many initiatives remain local or project-based and rely on motivated professionals without sustained organisational embedding, contributing to fragmented implementation and persistent variability across services.^24^, ^25^, ^26^

In the Netherlands, the 2016 NICE guideline informed the 2022 Quality Standard on Youth in Transition from Paediatric to Adult Care,^27^ which provides guidance for embedding structured transitional care in routine practice. Dutch transitional care is primarily organised within hospital-based specialty services, where responsibility for preparing and transferring adolescents generally rests with paediatric and adult healthcare teams. In the absence of a national implementation model, hospitals and specialties retain considerable autonomy in how transitional care is organised and delivered. The Quality Standard aims to support more consistent implementation by specifying core principles and essential interventions across the phases of the transition process (Box 1). It also describes additional interventions that can further strengthen transitional care once key interventions are in place. However, the Quality Standard cannot enforce the implementation of transitional care in healthcare organisations.

Box 1 **Core principles and key interventions from the Dutch Quality Standard.30**

**Table:** 

**Phase**	**Principles**	**Key interventions**
Before transfer	Early preparation of young people and their parents	Individual transition plan (ITP)
	Coordination and continuity	
During transfer	Coordination and continuity	Joint clinic (warm handover) between paediatric and adult services
		Multidisciplinary team meeting (Transition MDT)
After transfer	Integration into adult care	Extended first consultation in adult care
Throughout transition	Attention to self-management and psychosocial support	Individual transition plan (ITP)
	Active involvement and shared decision making with AYAs	Transition coordinator

**Fig. 1:**
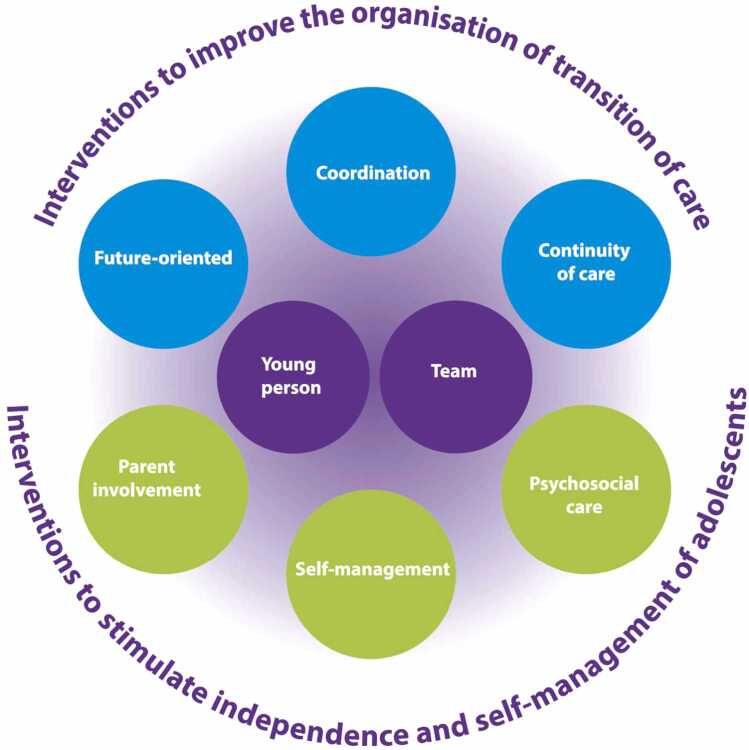
The ‘On Your Own Feet’ transition framework.^28^

Complementing this, the On Your Own Feet (OYOF) framework, developed through empirical research and expert consensus in 2008, conceptualises transitional care across eight principles that reflect key organisational and developmental components of high-quality transitional care (Fig. 1).^28^, ^29^ While the Quality Standard primarily organises transitional care according to the phases of the transition process,^30^ the OYOF framework adopts a cross-cutting perspective, structuring care into principles that span these phases. Collectively, these principles offer a comprehensive framework for examining how transitional care is organised and embedded across healthcare settings.

Still, insight into how everyday transitional care practices in Dutch university hospitals align with the Quality Standard and these OYOF principles across the full transition care pathway remains limited. Worthy of note in this regard is that in academic settings the care for AYAs with complex chronic conditions often involves multiple specialties and is embedded within intricate organisational structures. While many evaluations focus on a single condition, department, or transition phase, less is known about how transitional care is organised hospital-wide across specialties.

Against this background, the present study examines how transitional care for AYAs with chronic conditions is organised across medical specialties in paediatric and adult services in two Dutch university hospitals. Using a convergent mixed-methods design, we assess whether and how key transition interventions are implemented across the phases of preparation, transfer, and integration into adult care, and to what extent practices align with the Quality Standard and the eight OYOF principles. The ultimate goal is to identify organisational patterns and implementation determinants that can inform strategies for more sustainable and developmentally appropriate transitional care in academic hospital settings.

## 2 Methods

### 2.1 Study design

A convergent mixed-methods design was employed.^31^ Qualitative and quantitative data were collected within the same interview, analysed separately, and integrated during interpretation to capture both the implementation of transition interventions and the organisational and contextual factors shaping practice.

Data were collected at the level of the medical specialty through: (1) semi-structured interviews exploring routine practices, facilitators and barriers, and experiences across the phases of preparation, transfer, and post-transfer integration; and (2) a structured questionnaire assessing the presence of key transition interventions (see Appendix A). The questionnaire was administered verbally and cross-checked during the interview to support consistent interpretation.

### 2.2 Setting and participants

Data collection took place between January 2022 and January 2025 at Erasmus Medical Centre (Rotterdam) and University Medical Centre Utrecht (UMCU, Utrecht). All paediatric and adult specialty services providing care for AYAs with chronic conditions for whom transfer from paediatric to adult care occurs or is anticipated were approached.

The unit of analysis was the medical specialty, defined as the paediatric and/or adult care team within a specialty responsible for transitional care for a specific chronic condition. Specialties varied in their level of specificity, ranging from broad departments (e.g., neurology) to highly specialised, condition-focused services (e.g., Pompe disease or cystic fibrosis care). We aimed to include both paediatric and adult perspectives within each specialty; however, in some cases only paediatric professionals participated because adult-care colleagues were unavailable or did not respond despite repeated invitations. In some cases, a single professional or service covered both contexts. Within each specialty, one or two healthcare professionals involved in transitional care (e.g., nurse practitioners/physician assistants, nurses, or medical specialists) were nominated to participate. In total, 90 professionals representing 50 medical specialties were included. Because sampling prioritised broad coverage, no a priori sample size was determined.

### 2.3 Data collection

#### 2.3.1 Structured questionnaire

Participants completed a short, verbally administered questionnaire comprising closed-ended (yes/no) items assessing the presence of transitional care interventions within the medical specialty (see Appendix A). Responses were reviewed and clarified during the subsequent interview to ensure consistent interpretation.

The questionnaire was developed in an exploratory study on transitional care and self-management support conducted at Erasmus MC–Sophia Children’s Hospital, a Dutch academic paediatric hospital, and has since been applied in Dutch hospital-based studies evaluating transitional care practices.^10^, ^32^

#### 2.3.2 Semi-structured interviews

All participants completed a semi-structured interview (30–60 min), conducted either face-to-face or online (via Microsoft Teams). Four interviews were conducted with two participants. Interviews were conducted in Dutch, audio-recorded, and transcribed verbatim.

The interview guide was informed by the Quality Standard for Transition of Care^27^ and structured around three phases: preparation, transfer, and post-transfer integration. It was applied flexibly to allow exploration of additional experiences and perspectives as they emerged.^33^ Consistency across sites was ensured through a shared interview protocol, structured probing guidance, and standardised transcription procedures.

At Erasmus MC, one researcher (Rotterdam University of Applied Sciences) conducted seven interviews, and trained students conducted 34 interviews under close supervision of AvS (Professor Transitions in Care). Students received structured interview training, used standardised materials, participated in regular intervision sessions, and interview quality was periodically reviewed. The remaining Erasmus MC interviews were conducted by JS (n = 15). All UMCU interviews were conducted by MB (n = 33).

### 2.4 Data analysis

#### 2.4.1 Quantitative analysis

Questionnaire data were analysed descriptively at the medical specialty level using frequencies and percentages (IBM SPSS Statistics, version 28). Where appropriate, exploratory hospital comparisons were conducted using Fisher’s exact tests. Given the primarily descriptive aim of the study, p-values were interpreted cautiously (p < .05), without adjustment for multiple testing and without drawing causal inferences.

#### 2.4.2 Qualitative analysis

Interview transcripts and explanatory notes were analysed thematically.^34^ Initial coding was guided by a deductive framework based on the three phases of transition (preparation, transfer, post-transfer integration) and was supplemented by inductive coding to capture additional concepts emerging from the data. Codes relating to factors that influenced the implementation of transitional care were subsequently grouped into broader categories representing implementation determinants.

These determinants were interpreted using the Integrated Checklist of Determinants of Practice (TICD), informed by the Theoretical Domains Framework, developed by Flottorp et al.^35^ The TICD distinguishes seven domains influencing implementation: guideline-related factors, individual healthcare professional factors, patient factors, professional interactions, incentives and resources, capacity for organisational change, and social, political and legal factors. Furthermore, an expert panel in an unpublished two-round Delphi study conducted in 2020–2021 identified determinants across all seven TICD domains as highly relevant to transitional care implementation.

Factors described by participants as enabling or supporting the organisation or delivery of transitional care were classified as facilitators, whereas those described as hindering or constraining implementation were classified as barriers. Two researchers (JS, MB) independently coded transcripts, and discrepancies were discussed until consensus was reached. This process supported the credibility and trustworthiness of the analysis.^36^ Coding and data management were facilitated using ATLAS.ti (version 22).

#### 2.4.3 Mixed-methods integration and principle-based scoring

Integration followed a convergent design logic^31^: Questionnaire data were used to describe which interventions were reported as present, while interview data provided insight into how transitional care was organised and why practices varied across medical specialties.

In addition, principle-based scoring was applied to summarise the extent to which transitional care was embedded within each medical specialty. Using both interview and questionnaire data, each specialty was assessed against the eight principles of the OYOF framework and rated on a four-point scale (1 = usual care with minimal transitional care elements; 2 = ad hoc transitional care; 3 = structured transitional care not yet fully embedded in routine practice; 4 = fully embedded transitional care). Operational scoring criteria for each principle, including guiding considerations used during the assessment, are provided in Appendix B. This approach builds on earlier research assessing implementation levels across hospital settings.^10^

Two researchers reviewed all data from one of the two hospitals involved. Three other researchers independently scored selected medical specialties across both hospitals, resulting in at least two independent ratings per specialty. Final scores were established during calibration meetings through discussion aimed at reaching consensus. Interrater reliability of the initial ratings was assessed using intraclass correlation coefficients (ICC), while internal consistency across the eight principles was evaluated using Cronbach’s alpha. ICCs were calculated using a two-way random-effects model with absolute agreement, reporting both single-measure ICCs (for initial ratings) and average-measure ICCs (for consensus ratings). Final consensus scores were used in all subsequent analyses.

### 2.5 Ethical considerations

The study was approved by the Medical Ethics Review Committee of Erasmus MC (MEC-2014-246 addendum 2; 2021) and the Medical Ethics Review Committee of UMC Utrecht (MEC-U) (AW25.021/W24.057) and classified as not subject to the Medical Research Involving Human Subjects Act under Dutch regulation.

All participants provided written informed consent prior to participation, and consent for audio-recording was obtained before each interview. Participation was voluntary, and participants could withdraw at any time without consequence. Data were anonymised, stored securely, and handled in accordance with the EU General Data Protection Regulation and institutional policies. Participants were offered the opportunity to review their transcripts for accuracy.

## 3 Results

### 3.1 Quantitative results

#### 3.1.1 Study sample

Ninety healthcare professionals participated, representing 50 medical specialties (listed in Appendix C). Of the 50 specialties, 30 were from one hospital and 20 from the other. For 35 medical specialties, input was obtained from both paediatric and adult services. In the remaining specialties, a single clinician represented the specialty, either because one professional covered both paediatric and adult care (n = 8) or because additional adult-care colleagues were unavailable (n = 7). Overall, 47 participants (52%) were medical specialists and 43 (48%) were nursing professionals (nurse practitioners/physician assistants and nurses). Participant characteristics are summarised in Table 1.

**Table 1:** Characteristics of participating healthcare professionals and care settings.

	Erasmus MC (n = 57)	UMCU (n = 33)	Total (n = 90)
**Care setting**			
Paediatric care professionals	30 (58%)	22 (42%)	52 (58%)
Adult care professionals	27 (71%)	11 (29%)	38 (42%)
**Professional background**			
Medical specialists	22 (47%)	25 (53%)	47 (52%)
Nurse practitioners/Physician assistants	20 (74%)	7 (26%)	27 (30%)
Nurses	15 (94%)	1 (6%)	16 (18%)

#### 3.1.2 Implementation of transitional care interventions across phases

**Fig. 2:**
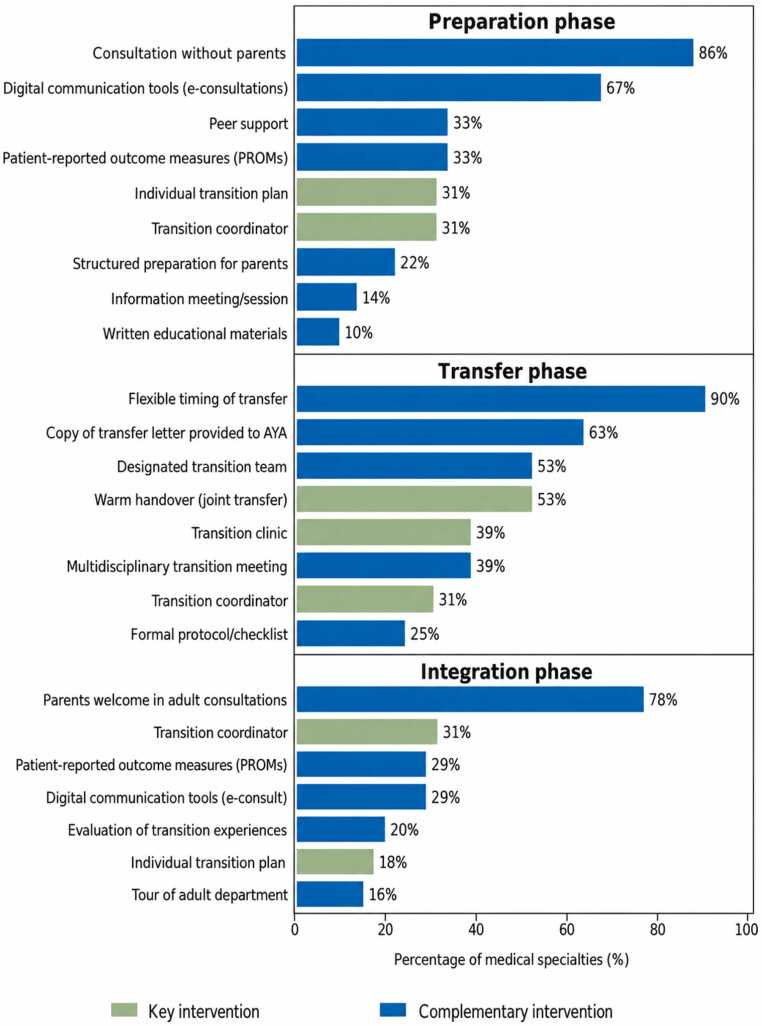
Adoption of transition interventions across all medical specialties by phase of transition.

Across the medical specialties, self-reported implementation of transitional care interventions varied across the three transition phases: preparation (paediatric care), transfer, and post-transfer integration (adult care) (Fig. 2).

In the preparation phase, 31% of specialties reported using an individual transition plan (ITP). This structured, personalised tool supports transition preparation, tracks progress and facilitates discussions about autonomy and self-management; the *Ready Steady Go* questionnaire is one example.^37^ During the transfer phase, 53% reported providing a warm handover, while 31% reported involvement of a transition coordinator. In the post-transfer integration phase, 18% reported continued use of an ITP after transfer. Fig. 2 further distinguishes between key interventions and complementary interventions, as defined in the Quality Standard.

#### 3.1.3 Exploratory comparisons between hospitals

Implementation patterns were broadly similar across both hospitals. However, formal protocols/checklists were reported more frequently at one hospital than at the other (Fisher’s exact p = 0.015). No statistically significant differences between hospitals were observed for any other interventions (all p > .10).

### 3.2 Qualitative results

Across interviews, healthcare professionals shared largely consistent views on what constitutes high-quality transitional care. They emphasised the importance of early, gradual preparation, developmentally appropriate support, continuity between paediatric and adult services, and fostering adolescent autonomy while involving parents when appropriate. These elements aligned with key principles of the OYOF framework. At the same time, professionals described substantial variation in how these principles were implemented in practice. Some specialties had formalised transition structures in place, whereas others relied on informal approaches that were applied inconsistently and often depended on individual professionals. To explore the factors underlying this variation, facilitators and barriers were mapped onto the seven-domain framework proposed by Flottorp et al.^35^ (Table 2a, Table 2b, Table 2c, Table 2d, Table 2e, Table 2f, Table 2g). Below, we summarise the dominant patterns across domains.

#### 3.2.1 Guideline-related factors (Table 2a)

Participants generally valued the Dutch Quality Standard and related tools but also noted that their presence alone was insufficient to achieve consistent implementation. Differences in interpretation, a lack of hospital-wide policy and reinforcement, and limited integration into routine workflows were frequently cited as reasons why guideline elements were not applied uniformly.

**Table 2a:** Guideline-related factors.

**Determinant**	**Facilitators**	**Barriers**	**Example quote**
**Clarity and availability of guideline**	• Structured care pathway is available as a model for other departments • Professionals widely acknowledge the value of national standards and tools	• Expectations of diagnosis-specific protocols • Absence of institution-wide vision • Conceptual ambiguity regarding key terms (divergent expectations and practices)	*“We have a clinical care pathway for the youth clinic: the transition clinic. It resembles a flowchart indicating what needs to be done at each stage and who is responsible.” (AC-EMC)*
**Applicability and integration of guideline into routine care**	• Ability to adapt guidelines and tools to local workflows and routines • A manageable number of tools and guidelines	• Insufficient integration in daily work process • Lack of shared interpretation across departments	*“PC and AC are often on different pages because they use different treatment protocols, and sometimes those protocols clash, which makes it hard for them to fully understand each other.” (AC-UMCU)*

*Note.* Quotations are labelled by setting (paediatric care [PC] vs adult care [AC]) and hospital (UMCU vs Erasmus MC [EMC]).

#### 3.2.2 Individual healthcare professional factors (Table 2b)

Professionals described variability in role clarity and ownership. Nurse practitioners were frequently identified as stable points of contact and (in)formal coordinators who supported continuity and contributed to service improvements. However, when transitional care relied on a small number of “champions”—particularly in the absence of standardised documentation or structured consultation routines—implementation was perceived as fragile and vulnerable to staff turnover and competing clinical priorities.

**Table 2b:** Individual healthcare professional factors.

**Determinant**	**Facilitators**	**Barriers**	**Example quote**
**Role clarity and ownership**	• (In)formal transition coordinators (often nurse practitioners) • Identified “transition champions” who drive improvement and innovation	• Informal, unstructured team coordination, risk of being unsustainable • Limited collective accountability within teams	*“It feels like we have to put in too much effort to get things done. We make an annual plan, but it gets postponed. Everyone says the moment is important, but not everybody is present.” (PC-EMC)*
**Skills, confidence, and documentation**	• Nurse practitioners as consistent contacts, focus on emotional and educational aspects of care • Flexibility to informally integrate transition related topics	• AC clinicians are underprepared and play a limited role • Lack of standardised consultation structure and poor documentation • Transitional care as a complex and overwhelming subject	*“Nurse practitioners are increasingly becoming 'case managers' who bring order to multidisciplinary care. They know the patients well and can therefore serve as consistent points of contact.” (PC-UMCU)*

*Note.* Quotations are labelled by setting (paediatric care [PC] vs adult care [AC]) and hospital (UMCU vs Erasmus MC [EMC]).

#### 3.2.3 Patient and parental factors (Table 2c)

Adolescent engagement and autonomy were widely seen as central to successful transition. Independent consultations without parents present and the use of youth-friendly communication tools, such as *Ready Steady Go*, were considered helpful, though their application was inconsistent. While parental involvement was recognised as important, participants frequently highlighted the absence of structured approaches to help parents gradually transfer responsibility or stimulate their child’s independence. In addition, the evaluation of transition experiences was typically informal and ad hoc, rather than a systematic practice.

**Table 2c:** Patient and parental factors.

**Determinant**	**Facilitators**	**Barriers**	**Example quote**
**Adolescent engagement and autonomy**	• Independent consultations as drivers for youth autonomy • Use of digital communication tools that align with young adult preferences	• Independent consultations limited to short, informal moments • Adolescent passivity (e.g., non-adherence) • Lack of tools or training on how to deal with age group	*“There is a kind of ‘infantilization of teenagers’ where decisions are still made for them. Young people easily fall into this role, which only perpetuates this dynamic.” (PC-UMCU)*
**Parental involvement**	• Growing recognition of parental empowerment • Preference for parental involvement during consultations	• Lack of structured parental guidance and step-back strategies	*“We could invest more in supporting parents. It’s evident that parents often find it difficult to let go. I believe we could take a step in guiding them through that process.”* *(PC-EMC)*
**Feedback and evaluation**	• Ad hoc follow-up by social workers to check in with adolescents about their transition experience	• No systematic collection of patient/parent experience data	*“I don’t evaluate it [transition experience], but I would like to hear it. I believe it’s going well but am not entirely sure because I did not ask them.” (AC-EMC)*

*Note.* Quotations are labelled by setting (paediatric care [PC] vs adult care [AC]) and hospital (UMCU vs Erasmus MC [EMC]).

#### 3.2.4 Professional interactions (Table 2d)

Collaboration between paediatric and adult care was identified as crucial for continuity. Warm handovers, joint consultations, and dual-role professionals who work across both paediatric and adult settings were seen as ways to strengthen collaboration and facilitate transfer. Conversely, limited collaboration and familiarity between paediatric and adult care teams, inconsistent information exchange, and loss to follow-up after transfer to another hospital were noted as barriers that weakened shared responsibility across the paediatric–adult boundary.

**Table 2d:** Professional interactions.

**Determinant**	**Facilitators**	**Barriers**	**Example quote**
**Collaboration and communication**	• Warm handovers / joint consultations as part of the transition process • Dual-role professionals bridging paediatric–adult care • Short communication lines between PC and AC	• Inconsistent use of warm handovers • Warm handovers seen as overkill for transferring patients smoothly • Loss to follow-up following transfer of care to another hospital	*“It’s also nice that the paediatrician is there, so adult care seems less daunting, and the patient can really be reassured by a familiar doctor.” (AC-UMCU)*
**Team culture and coordination**	• Transition clinics/group consultations • Transition MDTs as improvement for team feeling • Structured involvement of clinical geneticists	• Fragmented adult-care culture • Differences in working methods and organisation • Unclear responsibilities and redundancy with multidisciplinary care	*“Here in paediatric care, everything is very structured, and everyone talks to each other, but in adult care, they’re basically all on islands.” (PC-UMCU)*

*Note.* Quotations are labelled by setting (paediatric care [PC] vs adult care [AC]) and hospital (UMCU vs Erasmus MC [EMC]).

#### 3.2.5 Incentives and resources (Table 2e)

Participants associated the feasibility of implementation with time, tools, and infrastructure. Extended consultation time and flexible use of tools were seen as enablers. Some professionals noted that transition consultations could create opportunities to address broader life domains, including psychosocial wellbeing and participation in education or work, although these aspects were not consistently integrated into routine care. Barriers included a lack of protected time, limited integration of electronic health records and Information and communication technology (ICT) systems, and the absence of reminders and documentation routines, all of which hindered the consistent delivery of transitional care.

**Table 2e:** Incentives and resources.

**Determinant**	**Facilitators**	**Barriers**	**Example quote**
**Time and workload**	• Extended consultation time in adult care	• Insufficient time and resources to consistently apply tools such as the ITP or *Ready Steady Go*	*“In AC, I make sure to focus on the transition during the first consultation. I pay more attention to the personal situation and less to the medical aspects. I take a full hour to really get to know the person.” (AC-UMCU)*
**Tools and practical support**	• Flexible use of structured tools as conversation guidelines • Digital messaging/e-consults • Custom made tools fitting the patient population	• Lack of systematic tool use (hinders open dialogue, not always considered relevant for patient group) • Limited integration of electronic health records and ICT systems • No digital reminders for transition progress	*“What needs to improve, I think, is having a plan that outlines which topics to discuss at what age. Now, that’s still very vague.” (AC-EMC)*

*Note.* Quotations are labelled by setting (paediatric care [PC] vs adult care [AC]) and hospital (UMCU vs Erasmus MC [EMC]).

#### 3.2.6 Capacity for organisational change (Table 2f)

Medical specialties varied in their ability to sustain transitional care over time. Managerial support and flexible transfer windows were seen as beneficial, particularly for tailoring care to individual needs. Common constraints included staffing shortages, limited resources (including funding and workspace), and structural differences between paediatric and adult care models, which participants described as complicating continuity and collaboration.

**Table 2f:** Capacity for organisational change.

**Determinant**	**Facilitators**	**Barriers**	**Example quote**
**Organisational commitment and flexibility**	• Institutional commitment to transitional care • Flexible transfer window (16–22 years)	• Resource constraints (staffing, funding, workspace) • Shortened transfer windows due to capacity shortages • High workloads leaving no room for innovation	*“Some children with developmental delays may realistically need paediatric follow-up even after 18.” (PC-EMC)*
**Financial structures**	• Strategic timing of transitional care activities before age 18, when care is covered without patient charges	• Lack of reimbursement/billing codes for transition activities	*“Finances are a big problem—no billing code can be opened for this.” (AC-UMCU)*
**Care model differences**	• Occasional visit to the adult clinic (for example for an assessment) helps with getting acquainted	• Mismatch between supportive paediatric model and independent adult model (“culture shock”) • Physical distance between paediatric and adult care complicating collaboration	*“Transition between PC and AC is complex and leads to disappointment because the AC culture differs. Interventions are organised differently, causing tension and information loss at handover.” (PC-UMCU)*

*Note.* Quotations are labelled by setting (paediatric care [PC] vs adult care [AC]) and hospital (UMCU vs Erasmus MC [EMC]).

#### 3.2.7 Social, political and legal factors (Table 2g)

At a broader level, participants described how policy and governance shape prioritisation. While the Quality Standard established transitional care as a component of “good care”, many respondents pointed out the lack of hospital-wide policies that translate the standard into institutional strategy. Consequently, implementation was often viewed as dependent on individual professionals or short-term projects rather than being structurally integrated into the organisational structure.

**Table 2g:** Social, political and legal factors.

**Determinant**	**Facilitators**	**Barriers**	**Example quote**
**Policy and leadership**	• The Quality Standard legitimizes transition practices as components of good care • Active leadership/management support to help engage those reluctant to change	• Lack of institutional backing • No intermediate policy translating national guidelines to hospital level	*“There’s a Quality Standard and local departments, but the hospital-level commitment is missing. Without that layer, recognizing the urgency and embedding transition in policy, falls to individual caregivers — and progress is limited.” (PC-UMCU)*
**Governance and strategy**	• Shared responsibility across teams	• Symbolic/short-term projects without structural changes	*“I find it unfortunate that a lot of time is spent renaming things we already do. We give it a nice label, are satisfied, and close the project, while real issues persist. This also happens politically.” (AC-EMC)*

*Note.* Quotations are labelled by setting (paediatric care [PC] vs adult care [AC]) and hospital (UMCU vs Erasmus MC [EMC]).

Across domains, participants identified variation as primarily linked to how deeply transitional care was embedded as routine care, rather than to gaps in knowledge or motivation. Specialties with well-defined pathways, shared responsibilities, and established paediatric–adult collaboration reported more consistent practice and less reliance on individual efforts. In contrast, specialties lacking these structures were more vulnerable to staff turnover, resource constraints, and informal coordination.

Although facilitators and barriers were identified across all TICD domains, participants generally described barriers more consistently and in greater detail than facilitating factors. Limited organisational embedding, resource constraints, insufficient adult-care involvement, and reliance on individual professionals emerged as recurring challenges across specialties. Facilitators, such as transition coordinators, warm handovers, and established paediatric–adult collaborations, were also identified but were often described as local initiatives rather than routinely embedded practices. Box 2 synthesises these findings by outlining key facilitators and structural barriers to embedding transitional care into routine practice.

Box 2 **Key facilitating factors and structural barriers underlying transitional care implementation.**

**Table:** 

**Facilitating factors**	**Structural barriers**
National transitional care guidelines that legitimize and support local initiatives.	Absence of a hospital-wide transitional care policy translating national guidelines into institutional practice.
Availability of a clearly defined transition pathway specifying roles and responsibilities across PC and AC.	Unclear or inconsistently applied roles and responsibilities within multidisciplinary transition pathways.
Designated nurses or nurse practitioners acting as (in)formal transition coordinators.	Reliance on individual professionals due to lack of structurally embedded coordination roles.
Dual-role professionals spanning PC and AC, thereby supporting continuity and trust.	Limited involvement of adult care teams in transitional care planning and follow-up.
Individual, adolescent-centred care models, including independent consultations without parents.	Lack of structured attention to adolescents’ development of autonomy
Involvement of parents in the process of encouraging their child’s autonomy and independence	Lack of structured guidance to support parents in gradually transferring responsibility to their child.
Use of accessible communication tools aligned with adolescents’ digital preferences.	Inconsistent use of transition-specific tools, reflecting differing paediatric and adult care models regarding support and autonomy.
Structured paediatric–adult collaboration, such as warm handovers and joint consultations.	Limited information exchange between PC and AC, as transition-related information in the electronic health record is not systematically reviewed.
Team-based care models (e.g., transition clinics and multidisciplinary discussions).	Time constraints, funding limitations, and institutional policies that hinder sustained multidisciplinary collaboration.
Flexible transfer windows supported by local leadership and organisational commitment.	Lack of structural coordination and long-term organisational embedding, leading to dependence on individual initiatives.

### 3.3 Integrated scoring of transitional care principles (OYOF)

To move beyond simply identifying individual interventions, we integrated interview and inventory data, applying scoring based on the OYOF framework to assess the extent to which transitional care was embedded within each medical specialty. Internal consistency across the eight principles’ scores was excellent (Cronbach’s α = .91). Agreement between raters on the initial independent ratings was high when averaged across raters (average-measure ICC = .91), but lower for individual raters (single-measure ICC = .38). Discrepancies were resolved through consensus, and the final consensus scores were used for subsequent analyses.

Across medical specialties, total OYOF scores (the sum of eight principles rated 1–4) ranged from 8 to 27 (theoretical range 8–32). The median total score was 14.5 (IQR 12–20; P25 = 12, P50 = 14.5, P75 = 20). Based on empirical quartile cut-offs, specialties were grouped as follows: Q1 (≤12; n = 15), Q2 (13–14; n = 10), Q3 (15–18; n = 11), and Q4 (≥20; n = 14).

Qualitative interview findings indicated that specialties in the upper quartile more often reported a more organised approach across the principles. These specialties reported well-defined pathways, responsibilities, timelines, and stronger collaboration between paediatric and adult teams. Coordinating roles, often held by nurse practitioners, were more commonly described, as were warm handovers, joint consultations, and consultations without parents. In contrast, specialties in the lower quartiles often described transitional care as less standardised, with unclear responsibilities, less consistent involvement of adult-care clinicians prior to transfer, and limited or inconsistent use of structured tools such as ITPs and structured parental guidance.

**Fig. 3:**
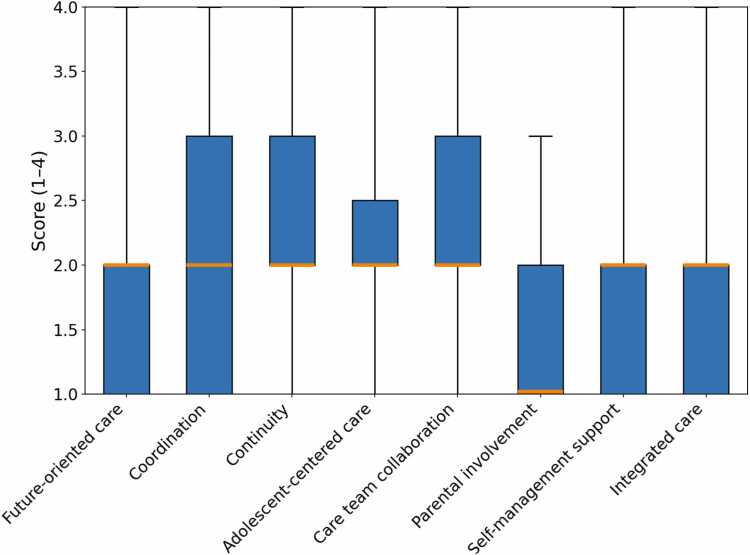
Distribution of principle-level scores (1–4) across the eight transitional care principles (median scores in orange).

To explore differences across specialties in relation to specific transition principles, Fig. 3 displays the distribution of scores for each OYOF principle (also see Appendix B). These scores were derived from the integrated analysis of interview and questionnaire data and assigned using a consensus-based scoring procedure.

Median scores were generally centred around 2 for most principles, with parental involvement having a lower median of 1. Principles related to coordination, continuity, and care team collaboration showed more favourable distributions, with scores more often extending into the higher range (3–4). Adolescent-centred care was positioned in the middle. In contrast, principles related to developmental preparation and support, including future-oriented care, self-management support, integrated care, and parental involvement, were more concentrated in the lower score range, with fewer reaching the highest scores. Specialties with higher scores for parental involvement also tended to have higher scores for adolescent-centred care and future-oriented care. Spearman correlation analyses (n = 50) revealed a strong positive association between parental involvement and adolescent-centred care (ρ = .71, p < .01) and a moderate-to-strong association between parental involvement and future-oriented care (ρ = .58, p < .01). In the scoring framework, higher parental involvement reflected structured support for parents in adapting their role and gradually transferring responsibility to the adolescent, rather than continued parental control over care. This may explain why parental involvement and adolescent-centred care tended to co-occur: specialties that explicitly supported parents in stepping back also more often supported adolescent autonomy, participation, and individual developmental needs. These patterns were consistent with qualitative findings, which highlighted limited shared ownership and a lack of consensus on how and when parents should gradually step back during the transition process.

Scores for continuity and care team collaboration were relatively higher and exhibited less variation across specialties compared to several other principles. In the interviews, these domains were typically described as established routines and coordination practices, particularly within paediatric services and in specialties with longstanding paediatric–adult collaboration across the transition boundary.

## 4 Discussion

### 4.1 Overall interpretation

Our findings suggest that professionals across the two university hospitals largely shared a common understanding of high-quality transitional care, encompassing early, gradual preparation, developmentally appropriate support, continuity across the paediatric–adult boundary, and appropriate parental involvement. However, these principles were not consistently applied. This indicates that the primary challenge lies in translating shared goals into sustained everyday practice. In this regard, our findings reinforce the view of transitional care as a developmental process where AYAs gradually build autonomy and self-management skills.^3^, ^5^, ^6^ They also underline the relevance of implementation frameworks, such as the TICD framework, which emphasises organisational and professional determinants—including role clarity, interprofessional collaboration, and resource availability—as essential conditions for sustained practice.^35^

### 4.2 Principal findings

Our findings further indicate that transitional care was not equally integrated across the care pathway. Practices during the preparation and transfer phases were more consistently incorporated into routine care, while post-transfer integration into adult services was less reliably implemented. The relative weakness of post-transfer integration highlights a broader issue: transition care delivery often remains focused on the period before and during transfer, rather than after it. This observation is consistent with evidence from previous syntheses.^8^, ^21^, ^22^ Nevertheless, international guidelines emphasise continued support after transfer as a core element of high-quality transitional care.^12^, ^16^, ^19^, ^27^

Differences between hospitals were limited, whereas there was substantial variation between medical specialties within hospitals. This suggests that implementation was primarily influenced at the specialty and care team level, rather than by hospital-wide structures. A plausible explanation is the limited presence of hospital-wide transitional care policies, reinforcement mechanisms, and formalised responsibilities, which left implementation largely dependent on specialty-level factors such as local leadership, workflow alignment, role clarity, team culture, and the degree of collaboration between paediatric and adult services.^35^ Existing literature on hospital-wide transitional care implementation has mainly focused on programme development and system building rather than on comparative evaluation across departments.^38^, ^39^, ^40^, ^41^ This interpretation is also supported by a recent institution-wide mixed-methods assessment of healthcare transition in a tertiary children’s hospital, which reported substantial heterogeneity in healthcare transition practices within and between divisions and care programmes and highlighted the absence of a coordinated institutional approach.^42^ Together, these findings suggest that, in the absence of strong hospital-wide governance and reinforcement, transitional care remains largely organised at the local level, leading to considerable specialty-level variation across departments.

Although this study did not assess inequities according to patient-level characteristics, the substantial variation between medical specialties may indicate potential service-level inequities in access to structured transition support. In practice, AYAs’ access to coordinated and developmentally appropriate transitional care may depend on the specialty team in which they receive care, the presence of coordinating roles, and the degree to which paediatric–adult collaboration is integrated into usual care.

To capture the extent to which transitional care was embedded into routine practice, we combined interview and questionnaire data with scores across the eight OYOF principles.^27^, ^28^ Although continuity and care team collaboration scored relatively higher than developmental support, overall implementation remained modest across all principles. Interview finding indicated that continuity and collaboration more often aligned with existing coordination routines, particularly in paediatrics and specialties with longstanding paediatric–adult collaboration, whereas developmental components were less structured and more reliant on individual initiatives. The positive association between parental involvement and adolescent-centred care suggests that these principles were not implemented as opposing approaches but tended to co-occur in specialties with a more developmentally oriented transition approach. In this context, parental involvement refers to supporting parents in adapting their role and gradually transferring responsibility to the adolescent, thereby reinforcing rather than limiting adolescent autonomy and participation. However, relatively higher scores in these areas do not imply consistent integration into routine care. Combined with the limited implementation of key transition interventions, these findings highlight substantial room for improvement in transitional care delivery.

Using the Flottorp framework clarified why shared endorsement of transitional care did not translate into routine delivery.^35^ Standards and local pathways were considered beneficial, but interpretations of what this entails differed, and key elements were not consistently integrated into daily workflows. Ownership and role clarity varied across teams. Nurse practitioners often acted as points of contact and informal coordinators, consistent with transition coordinator roles and nurse-led initiatives.^26^, ^43^, ^44^ However, person-dependent coordination remained vulnerable to staff turnover and competing priorities. Cross-boundary collaboration was widely seen as pivotal for continuity but was not consistently applied due to practical constraints. Together, inconsistent role clarity, person-dependent coordination, and practical barriers to cross-boundary collaboration help explain uneven implementation, in line with implementation theory and recent Dutch work on organisational embedding in academic hospitals.^35^, ^41^

Structured strategies to support parents in gradually transferring responsibilities to their child were largely absent. This is concerning, because parental roles and expectations shape opportunities for their children’s self-management practice,^5^, ^7^ and parent-focused intervention studies highlight both the need for such support and the challenges of embedding it in routine care.^45^, ^46^ Systematic evaluation of transition experiences and routine use of PROMs in adult care were also limited, consistent with broader evidence that measurement and evaluation remain underdeveloped in transitional care.^21^ Although approaches to readiness assessment remain heterogeneous,^23^ our findings primarily indicate limited routine outcome monitoring and post-transfer evaluation. Digital tools such as e-consultations may help support continuity, but their use in adult care appeared limited and dependent on infrastructure and workflow fit.^47^

### 4.3 Implications

Our findings indicate that transitional care remained largely organised at the level of individual specialties, with implementation reliant mainly on local initiatives. Improvement efforts should therefore explicitly address areas that were less consistently implemented across the hospitals involved in this study, particularly post-transfer support in adult care and developmentally oriented components such as self-management support and parental guidance.

Broader system-level work highlights the value of fostering shared understanding and coordinated improvement among stakeholders involved in healthcare transition.^48^ Our findings suggest that, within academic hospitals, such efforts may need to be complemented by more explicit, hospital-wide structures. Structured models—such as the Blueprint proposed by Buijs et al.,^41^ which integrates paediatric-adult collaboration, joint consultations, a transition coordinator, and regularly updated individual transition plans—may help reduce variation between specialties and support more consistent, developmentally appropriate transitional care.

### 4.4 Strengths and limitations

A key strength of this study is the convergent mixed-methods design, which enabled triangulation of intervention inventory data, qualitative findings, and integrated principle-based scores.^31^ This system-level approach captured not only whether transition interventions were in place, but also how consistently core principles were embedded in routine care. Rigour was strengthened through established thematic analysis procedures and consensus-based scoring.^34^, ^36^ A further strength is the involvement of a large number of medical specialties within two academic medical centres. Academic hospitals care for relatively large populations of adolescents with chronic and often complex conditions and rely on multidisciplinary collaboration, making them particularly relevant settings in which to examine how transitional care is organised and implemented.

Several limitations should be considered. First, findings reflect healthcare professionals’ perspectives only; without input from patients and parents, aspects of everyday care delivery may have been overlooked or portrayed more positively.^49^ In addition, no patient-level sociodemographic or clinical data were collected. Therefore, this study cannot determine whether transition support differed according to characteristics such as socioeconomic position, ethnicity, language proficiency, health literacy, gender, or disease complexity. Some specialties were represented by participants from either paediatric or adult care only, which limited our ability to capture both sides of the transition process and may have resulted in an incomplete picture of collaboration and continuity across the care pathway. Participation was voluntary, which may have introduced selection and response biases.^50^ Furthermore, the structured questionnaire assessed the presence of transition interventions using dichotomous (yes/no) response options. As a result, the measure did not capture variation in the extent, consistency, or maturity of implementation across specialties. Some interventions may have been applied only occasionally or to selected patient groups, while others may have been routinely embedded in practice, despite receiving the same response category.

Data collection procedures also differed between sites. At one site, interviews were conducted by several trained student interviewers under supervision, whereas at the other all interviews were conducted by a single experienced researcher. Although training and supervision aimed to ensure consistency, differences in interviewing experience and style may have influenced the depth and focus of the interviews and, consequently, the comparability of findings between sites.

Moreover, the specialties included ranged from broad departments to narrowly defined, condition-specific services (Appendix C). Consequently, implementation levels may partly reflect differences in organisational scale and focus rather than differences in commitment to transitional care, limiting the direct comparability of scores across specialties.

Data collection spanned a three-year period, during which organisational routines, staffing, resources, and transition practices may have changed within specialties. The study design did not allow these temporal changes to be systematically assessed, and the findings should therefore be interpreted as a snapshot of implementation during this period rather than as a reflection of stable practices over time. Furthermore, the cross-sectional design limits conclusions about causality and long-term sustainability, and comparisons between hospitals and medical specialties should be interpreted cautiously.

### 4.5 Conclusion

In the early implementation phase of the 2022 Quality Standard, transitional care interventions were present but varied substantially across medical specialties in two Dutch university hospitals. Overall uptake of key transition interventions remained limited, with most reported in only about one-third of specialties, indicating that structured transitional care had not yet been routinely established in most teams. Practices focused on preparation within paediatric care were more commonly in place than those supporting post-transfer integration into adult care. Development-oriented components were less consistently implemented than those related to continuity of care and care team collaboration. Variation across specialties exceeded differences between hospitals, suggesting that implementation was shaped primarily at the level of local organisation and team practices. More consistent implementation was observed in specialties with established coordination routines and active paediatric–adult collaboration, whereas limited role clarity, lack of structured processes, and reliance on individual professionals hindered routinisation. These findings indicate that sustainable implementation requires not only clearer coordination roles and more routine collaboration between paediatric and adult services, but also stronger organisational embedding beyond individual initiatives.

## CRediT authorship contribution statement

**Jobert Sturm:** Conceptualization, Methodology, Investigation, Formal analysis, Validation, Writing – original draft, Writing – review & editing. **Myrthe Braunstahl:** Conceptualization, Methodology, Investigation, Formal analysis, Validation, Writing – original draft, Writing – review & editing. **Heidi van Heijningen-Tousain:** Formal analysis, Validation, Writing – review & editing. **Marieke van Summeren:** Methodology, Validation, Supervision, Resources, Writing – review & editing. **Jane Sattoe:** Conceptualization, Methodology, Formal analysis, Validation, Funding acquisition, Supervision, Writing – review & editing, Project administration. **Johanna C. Escher:** Conceptualization, Methodology, Supervision, Resources, Writing – review & editing. **AnneLoes van Staa:** Conceptualization, Methodology, Formal analysis, Validation, Funding acquisition, Supervision, Writing – review & editing, Project administration.

## Ethical

We certify that this research complied with ethical standards, following the Helsinki Declaration (1975, revised in 2013) and relevant national regulations.

## Funding

This study is part of the ‘Transition Continued’ Project, funded by the Taskforce for Applied Research SIA, part of the Dutch Research Council (Grant no.: RAAK.PRO05.057), and by a doctoral grant from Rotterdam University of Applied Sciences awarded to Jobert Sturm.

## Declaration of Competing Interest

The authors declare that they have no conflicts of interest.

## Figures and Tables

**Table:** 

1. Congenital Cardiology
2. Cardiac Electrophysiology
3. Cerebral Palsy
4. Surgical Long-Term Follow-Up
5. Cystic Fibrosis
6. Dermatology
7. Hereditary congenital anomalies and disorders
8. Endocrinology
9. Reproductive Medicine / Fertility Care
10. Heart Transplantation
11. Haematology (Haemophilia)
12. Haematology (Sickle Cell Disease)
13. Inflammatory Bowel Disease
14. Infectious Diseases
15. Oral and Maxillofacial Surgery (Craniosynostosis)
16. Otorhinolaryngology (ENT)
17. Gastroenterology and Hepatology
18. Nephrology (Nephrotic Syndrome)
19. Nephrology (Transplantation and Dialysis)
20. Neurosurgery
21. Neurology (Neurofibromatosis Type 1)
22. Neurology (Neuromuscular Disorders & Tuberous Sclerosis Complex)
23. Orthodontics (Cleft Lip and Palate)
24. Orthopaedics (Scoliosis)
25. Rheumatology (Juvenile Idiopathic Arthritis)
26. Rheumatology
27. Rehabilitation Medicine
28. Home Mechanical Ventilation
29. Urology
30. Pompe Disease

**Table:** 

1. Home Mechanical Ventilation
2. Otorhinolaryngology (ENT)
3. Cleft Lip and Palate
4. Nephrology
5. Rheumatology
6. Cardiology
7. Pain Management / Pain Medicine
8. 22q11.2 Deletion Syndrome
9. General surgery
10. Gynaecology
11. Neuromuscular Disorders
12. Neurology
13. Immunology
14. Gastroenterology
15. Allergology
16. Rehabilitation Medicine
17. Clinical Genetics
18. General Paediatrics (including Spina Bifida)
19. Haematology
20. Orthopaedics

## Data Availability

Data will be made available on request.

## Appendix A Structured intervention inventory

**Transition Care Inventory Template**

**Specialty:**

**I. Background Information**

1. Which outpatient clinic or diagnostic group does this questionnaire refer to?
2. ___________________
3. Name of respondent 1: ___________________
  □ Paediatric care
  □ Adult care
  □ Other, namely: ___________________
4. Position of respondent 1: ___________________
5. □ Nurse consultant
6. □ Nurse specialist
7. □ Medical specialist, namely: ___________________
8. □ Other, namely: ___________________
9. Name of respondent 2: ___________________
  □ Paediatric care
  □ Adult care
  □ Other, namely: ___________________
10. Position of respondent 2: ___________________
11. □ Nurse consultant
12. □ Nurse specialist
13. □ Medical specialist, namely: ___________________
14. □ Other, namely: ___________________

**II. Patient Population**

6a. Paediatric care:

Approximate number of adolescents aged > 12 years treated by your team:

Number: ___________________

6b. Adult care:

Approximate number of young adults aged 18–25 years treated by your team:

Number: ___________________

7a. Paediatric care:

How many adolescents leave Paediatric care each year? (approximate average).

Number: ___________________

7b. Paediatric care:

To whom are these adolescents transferred? (multiple answers possible)

□ Adult care at own hospital

□ Adult care at other hospitals

□ General practitioner

□ Within the same expert center (no transfer)

8a. Adult care:

How many young people do you receive annually from Paediatric care or other sources?

Number: ___________________

8b. Adult care:

Where do these young people come from? (multiple answers possible)

□ Children’s hospital

□ Other hospitals/new patients

□ Within the same expert center (no transfer)

9. How would you rate the current collaboration between Paediatric and adult care for this patient group? Score (1–10)

Respondent 1: ___________________

Respondent 2: ___________________

**III. Collaboration Between Paediatric and Adult Care/Transfer**

10. Is there a fixed transition team of physicians, nurses, or nurse specialists who collaborate or meet regularly?

□ Yes

□ No

If yes, how is it composed? ___________________

11. At what age does the transfer from Paediatric to adult care usually take place?

Age: _______

12. Is flexible transfer possible (e.g., earlier or later than 18 years)?

□ Yes

□ No

13. Are adolescents involved in deciding to whom their care will be transferred?

□ Yes

□ No

14. Are adolescents involved in deciding the timing of the transfer?

□ Yes

□ No

15. Is a written transfer summary always prepared and/or received?

□ Yes

□ No

16. Do adolescents receive a copy of the referral or transfer letter?

□ Yes

□ No

17. Who schedules the first appointment in adult care?

□ Someone from the Paediatric team

□ The young person themselves

□ Other, namely: __________

18. What happens if the young person does not attend the first appointment or if the appointment is not scheduled? ___________________

19. Is a transition clinic organised?
  □ Yes
  □ No
  If yes: What does it involve? ___________________
  • Is a *warm handover* organised (i.e., a joint consultation between Paediatric and adult care)?
  • How often do joint consultations take place (once or multiple times)?
  • Where is the transition clinic organised?

20. Is a transition multidisciplinary team meeting (Transition MDT) organised?
21. (i.e., a joint patient discussion between Paediatric and adult care teams prior to transfer)

□ Yes

□ No

21. Is there a step-by-step plan or checklist for the transition/transfer process (e.g., a care pathway or protocol)?

□ Yes

□ No.

If yes, could a copy be shared? ___________________

22. Has a transition coordinator been appointed?

□ Yes

□ No

If yes, where is the coordinator positioned? ___________________.

□ Within Paediatric care

□ Within adult care

□ In both teams

23. Who fulfils the role of transition coordinator?

□ Nurse/Nurse specialist

□ Physician/Specialist

□ Other, namely: __________________

24. Is there interest in gaining insight into the experiences of young people who have undergone the transition? For example by:

• Using a validated questionnaire (OYOF-TES – *On Your Own Feet Transition Experiences Scale*, 20 questions)
• Organising a reflection session with several young people

___________________

Support can be provided for both approaches.

**IV. Preparation for Transition (Only ask Paediatric care)**

25. Is written information material about transition/transfer available?

□ Yes

□ No

If yes:

• Could a copy be shared? ___________________
• At what age is this information provided? ___________________

26. Is an educational meeting organised about transition for adolescents and their parents?

□ Yes

□ No

27. Are there activities where young people can meet peers? For example through a peer buddy who has already experienced the transition.

□ Yes

□ No

28. Is an individual transition plan used during preparation? (A systematic approach to gradually prepare adolescents and parents for greater independence and self-management.) Examples:

• Ready Steady Go
• Groei-wijzer

□ Yes

□ No

29. Are there specific activities aimed at parents to prepare them for their changing role during and after transition?

□ Yes

□ No

30. From the age of 16, are adolescents routinely given the opportunity to speak with healthcare professionals alone for part of the consultation?

□ Yes

□ No

31. How do you address psychosocial aspects of living with a chronic condition?

___________________

32. Do you use Patient-Reported Outcome Measures (PROMs)?

□ Yes

□ No

If yes:

• How are the results discussed? ___________________
• Which PROMs are used (e.g., core set of generic PROMs)? ___________________
• How are they administered (patient portal, KLIK system)? ___________________
• Are results discussed during consultations? ___________________

33. Do you use the “Beter Dichtbij” communication app in Paediatric care?

□ Yes

□ No

34. Are there other interventions or approaches used in the care of adolescents or in preparation for adult care? Examples:

• Serious game
• Knowledge tests
• Self-management web tools

___________________

**V. Follow-up/Reception in Adult Care (Only ask adult care)**

35. Is a tour of the new outpatient clinic or department organised for the young person?

□ Yes

□ No

36. Is an individual transition plan used in adult care? Examples:

• Groei-wijzer
• Ready Steady Go

□ Yes

□ No

37. Are transition experiences of young people evaluated?

□ Yes

□ No

If yes, how? ___________________

38. Are parents still welcome during consultations in adult care?

□ Yes

□ No

If yes, what is their role? ___________________

39. Are Patient-Reported Outcome Measures (PROMs) used in adult care?

□ Yes

□ No

If yes:

• Which PROMs? ___________________
• How are results discussed? ___________________
• How are they administered? ___________________

40. Do you use the “Beter Dichtbij” communication app in adult care?

□ Yes

□ No

41. How do you address psychosocial aspects of living with a chronic condition in adult care? ___________________

42. Are there other interventions or approaches used in the care of young adults? Examples:

• Serious game
• Knowledge tests
• Self-management tools

___________________

**Additional comments**.

___________________

## Appendix B Scoring criteria for assessment of transitional care practices

For each core principle (aspect of transitional care), assess the extent to which the team/department best reflects current practice on a scale from 1 to 4:

1. There is little or no specific attention to this aspect of transitional care within the team (use this score when no explicit focus on this core principle is present, or when only usual care is provided).
2. There is some attention to this aspect of transitional care within the team; however, substantial improvement is still possible.
3. There is considerable attention to this aspect of transitional care; the team is on the right track, but further improvement is still possible.
4. The team performs well on this aspect of transitional care; further improvement is not necessary, although continued attention remains important (use this score if the team could serve as a good practice example for this core principle).

**I. Organisation of the transition process**

**Principle A: Future-oriented care**

A written protocol or policy helps to organise transition in a future-oriented and planned manner. Starting preparation in a timely way (before the age of 14) and gradually working towards increasing responsibility and independence for the adolescent are key elements, as is familiarisation with adult care providers prior to transfer.

**When assigning a score, consider for example:**

• Whether a transition protocol or care pathway is used.
• Whether preparation for the transition process starts early with adolescents and parents.
• Whether this preparation is carried out in a structured manner.
• Whether the transition protocol and preparatory steps are known to all team members as well as to adolescents and parents.
• Whether future plans and challenges for the adolescent are regularly discussed during consultations.

**Principle B: Coordination**

The appointment of a transition coordinator who oversees the process (including communication and coordination between paediatric and adult care providers, and logistical aspects of transition and transfer) and who serves as a point of contact for adolescents and their parents is recommended.

**When assigning a score, consider for example:**

• Whether a designated transition coordinator is present within the team and acts as a point of contact for professionals and adolescents/parents.
• Whether there are professionals who work on both sides of the transfer (paediatric and adult care).
• Whether coordination between paediatric and adult care regarding transfer is in place.
• Whether the transition pathway is clear to adolescents, parents, and team members.

**Principle C: Continuity**

A shared transition policy, high-quality information transfer (both verbal and written), clarity within paediatric care about to whom the adolescent is transferred, and monitoring and evaluation of follow-up contribute to continuity of care during transition. A “warm handover,” in which paediatric and adult care providers jointly see the adolescent and parents, is a suitable intervention.

**When assigning a score, consider for example:**

• Whether the adolescent has a designated primary provider in both paediatric and adult care.
• Whether a warm handover takes place.
• Whether warm handovers also occur when adolescents are transferred to another institution.
• Whether coordination exists between paediatric and adult care regarding transfer and treatment.
• Whether continuity of treatment is ensured.
• Whether attention is paid to adolescents who are lost to follow-up.
• Whether evaluation and follow-up take place after transfer.

**II. Collaboration between adolescent and care team**

**Principle D: Adolescent-centred care**

Transition should be tailored to the individual situation and developmental stage of the adolescent. Their wishes, needs, and preferences should be explored and taken seriously. Adolescents should be actively involved in their own care and encouraged to participate in decision-making. One approach is seeing adolescents partly alone during consultations (e.g. from age 16), with parents joining later.

**When assigning a score, consider for example:**

• Whether adolescents are actively involved in decisions regarding treatment and transfer to adult care.
• Whether team members are aware of legal changes in decision-making authority (e.g. between ages 12–16 and ≥16 years).
• Whether adolescents and parents are informed about these changes.
• Whether adolescents are (partly) seen alone during consultations as part of policy rather than ad hoc practice.
• Whether adolescents’ wishes and preferences are sufficiently addressed in routine care.
• Whether care is adapted to developmental age in both paediatric and adult care.
• Whether adolescents have a voice in the design of the transition programme.
• Whether adolescents are involved in evaluation of transitional care.
• Whether PROMs results are discussed during consultations.
• Whether attention is paid to peer contact.

**Principle E: Care team collaboration**

Interdisciplinary coordination and collaboration between paediatric and adult care providers, alignment of working methods and procedures, and meeting new providers prior to transfer are essential for high-quality transitional care. Multidisciplinary transition meetings can support this process.

**When assigning a score, consider for example:**

• The extent to which paediatric and adult care providers know each other personally.
• Whether protocols, procedures, and working methods are aligned across paediatric and adult care where possible.
• Whether adolescents and parents meet adult care providers prior to transfer (e.g. during a transition clinic or warm handover).
• Whether adolescents and parents are encouraged to visit the adult care setting in advance.
• Whether an extended first consultation in adult care is offered to support relationship building.
• Whether multidisciplinary transition meetings are organised.
• Whether team members are motivated to initiate and sustain improvement efforts in transitional care.

**III. Preparation for self-management and independence**

**Principle F: Parental involvement**

Parents should remain involved during transition and receive guidance in gradually transferring responsibility and autonomy to their adolescent. Use of an individual transition plan (e.g. Ready Steady Go) supports this process. Adequate information for parents helps prevent negative expectations regarding transfer to adult care.

**When assigning a score, consider for example:**

• Whether sufficient attention is given to parental concerns and needs before and after transfer.
• Whether changing parental roles are addressed during consultations.
• Whether parents are welcomed in adult care.
• Whether parents are appropriately involved in both paediatric and adult care.
• Whether individual transition plans address changing parental roles.
• Whether parents and adolescents receive adequate information about the transition pathway and differences between paediatric and adult care.
• Whether there is a strong collaborative relationship between parents and professionals.

**Principle G: Self-management support**

A person-centred approach is essential to support adolescents in developing independence and self-management skills. Attention should be given not only to medical aspects but also to psychosocial challenges. Individual transition plans support structured development of self-management and transition readiness.

**When assigning a score, consider for example:**

• Whether preparation for independence and self-management starts early.
• Whether individual transition plans support gradual development of autonomy.
• Whether self-management and coping skills receive attention in both paediatric and adult care.
• Whether everyday challenges related to the condition are discussed during consultations.
• Whether illness beliefs and mental health are addressed.
• Whether professionals demonstrate understanding of difficulties adolescents may experience with self-management.

**Principle H: Integrated care**

Adolescents face challenges across multiple life domains during transition to adulthood. A holistic, integrated approach is therefore required, including attention to psychosocial wellbeing, quality of life, and participation. Sensitive topics (e.g. sexuality, substance use) should be addressed, and referrals made when needed.

When assigning a score, consider for example:

• Whether consultations address social participation (education, transport, work, leisure) in both paediatric and adult care.
• Whether mental health is systematically addressed or screened.
• Whether sensitive topics (e.g. smoking, alcohol, drugs) are discussed (e.g. using HEADSS screening).
• Whether advance care planning or future health risks are addressed where relevant.
• Whether access to psychological support or peer activities is offered.
• Whether professionals collaborate with schools, welfare organisations, youth care, or other external services when needed.

## Appendix C List of included medical specialties

1. **Erasmus MC**

2. UMCU
